# The role of unhealthy lifestyles in the incidence and persistence of depression: a longitudinal general population study in four emerging countries

**DOI:** 10.1186/s12992-017-0237-5

**Published:** 2017-03-20

**Authors:** Maria Cabello, Marta Miret, Francisco Felix Caballero, Somnath Chatterji, Nirmala Naidoo, Paul Kowal, Catherine D’Este, Jose Luis Ayuso-Mateos

**Affiliations:** 1 Centro de Investigación Biomédica en Red de Salud Mental. CIBERSAM, Instituto de Salud Carlos III, Madrid, Spain; 2 Department of psychiatry, Universidad Autónoma de Madrid, Madrid, Spain; 3 Department of Health Statistics and Information Systems, World Health Organization, Geneva, Switzerland; 4 Research Centre for Generational Health and Ageing, Faculty of Health, University of Newcastle, Newcastle, NSW Australia; 5 National Centre for Epidemiology and Population Health (NCEPH), Australian National University, Canberra, ACT, Australia; 6 Hospital Universitario de La Princesa, Instituto de Investigación Sanitaria Princesa (IIS Princesa), Diego de Leon, 62, Madrid, 28006 España

**Keywords:** Depression, Longitudinal study, Middle-income countries, Lifestyles

## Abstract

**Background:**

Unhealthy lifestyles and depression are highly interrelated: depression might elicit and exacerbate unhealthy lifestyles and people with unhealthy lifestyles are more likely to become depressed over time. However, few longitudinal evidence of these relationships has been collected in emerging countries. The present study aims i) to analyse whether people with unhealthy lifestyles are more likely to develop depression, and ii) to examine whether depressed people with unhealthy lifestyles are more likely to remain depressed. A total of 7908 participants from Ghana, India, Mexico and Russia were firstly evaluated in the World Health Organization’s Study on Global AGEing and Adult Health (SAGE) Wave 0 (2002–2004) and re-evaluated in 2007–2010 (Wave 1). Data on tobacco use, alcohol drinking and physical activity, were collected. Logistic regressions models were employed to assess whether baseline unhealthy lifestyles were related to depression in Wave 1, among people without 12-month depression in Wave 0 and any previous lifetime diagnosis of depression, and to 12-month depression at both study waves (persistent depression).

**Results:**

Baseline daily and non-daily smoking was associated with depression in Wave 1. Low physical activity and heavy alcohol drinking were associated with persistent depression.

**Conclusions:**

Unhealthy lifestyles and depression are also positively related in emerging countries. Smoking on a daily and non-daily basis was longitudinally related to depression. Depressed people with low physical activity and with heavy drinking patterns were more likely to become depressed over time. Several interpretations of these results are given. Further studies should check whether a reduction of these unhealthy lifestyles leads to lower depression rates and/or to a better clinical prognosis of depressed people.

## Background

According to World Health Organization (WHO), tobacco use, excessive alcohol drinking and physical inactivity are important modifiable risk factors for mortality and burden in the world [1]. These factors have been traditionally studied in the field of non-communicable diseases such as cancer, diabetes or heart attacks [2]. However, unhealthy lifestyles are also related to mental health [3]. Particularly, people with depression frequently smoke, have a higher prevalence of excessive alcohol drinking and are more physically inactive [4]. The relationships between depression and unhealthy lifestyles are probably reciprocal. Firstly, depression might elicit and exacerbate unhealthy lifestyles. Depression is related to the onset of physically inactivity [5], to the development alcohol use disorders [6] and to an increase in the risk of progression to daily smoking [7]. Some authors argue that depression drives to unhealthy lifestyles because people with depression use them as a “self-medication” to ameliorate depressive symptoms [7]. However, unhealthy lifestyles have been also longitudinally associated with depression. People who smoke [8] and drink excessively [9] are more likely to become depressed over time. This could be explained by the existence of common vulnerable factors independently associated with depression and with unhealthy lifestyles [10, 11]. However, a more direct relationship between some unhealthy lifestyles and depression has been also suggested. For example, tobacco might impact on some biological mechanisms that could increase the likelihood of developing depression [7, 12].

Existing evidence might also support that once unhealthy lifestyles and/or depression are present they might exacerbate one another. A chronic course of depression has been associated with an incidence of excessive alcohol use and with an increase in cigarette smoking [5] whereas, depressed people who smoked [8] were more likely to remain depressed over time.

A previous study reported that the highest prevalence of unhealthy lifestyles was found in concurrent depressed people, followed by lifetime depressed and never depressed [4]. However, it remains unclear whether a higher prevalence of unhealthy lifestyles is found in people with persistent depression compared to incident depression.

In contrast to the results reported in the Western literature, one study from China [13] found that smokers were less likely to develop and maintain depressive symptoms over time and that alcohol drinkers were at lower likelihood for developing depression. No other longitudinal studies to our knowledge, have studied the longitudinal relationship between unhealthy lifestyles and depression in emerging countries where depression is also related to poor population health measures, such as years lived with disability [14].

The study of lifestyles and their relationship with depression is particularly important in the middle-income countries. Firstly, because their economic growth has facilitated a rapid spread of unhealthy lifestyles, but national health policies have not responded as quickly to efficiently tackle them [15]. Secondly, because the relationship between unhealthy lifestyles and depression might work differently in emerging countries than in high income countries, since unhealthy lifestyles might be associated with different social circumstances; For example, high level of physical activity is associated with higher level of education in high income countries, whereas the opposite direction might be found in emerging countries [16], and finally, these factors can be modifiable by community-based interventions [17] which are convenient actions in the particular context of emerging countries, where the specialized mental health care coverage is still sparse [18].

Therefore, the present study aims to fill a gap in the literature by determining i) whether people with incident depression and persistent depression significantly differ in their prevalence of unhealthy lifestyles ii), whether people with unhealthy lifestyles are more likely to develop depression, and finally iii) whether people with depression and unhealthy lifestyles are more likely to remain depressed over time.

## Methods

### Design

The data are from four of the countries participating in the World Health Organization’s Study on Global AGEing and Adult Health (SAGE), a multi-country longitudinal study. These countries have been all classified as middle-income countries according to the World Bank classification [19].

### Sample and study procedures

SAGE study collected nationally representative samples of adults aged 50 years and older, and a smaller comparative sample of those 18–49 years in age. SAGE Wave 0 [20] was conducted between 2002–2004 and Wave 1 between 2007–2010. A subsample of SAGE Wave 0 respondents were revisited in SAGE Wave 1 in Ghana, India, Mexico, and the Russian Federation, resulting in a longitudinal data set of 7908 respondents in total. In India, all respondents aged 50 years and older were revisited in Wave 1 (*n* = 4888). In Ghana (*n* = 544), Mexico (*n* = 1876), and the European region of the Russian Federation (*n* = 600), a sub-set was randomly selected for the follow-up. The data from Waves 0 and 1 were linked based on unique household and individual identifiers, location, age and sex. Individual response rates for SAGE Wave 0 were 95% in Ghana, 94% in India, 100% in Mexico and 99% in the Russian Federation. Response rates for SAGE Wave 1 were 84% in the Russian Federation, 81% in Ghana and 68% in India and 53% in Mexico. The questions were translated into the local languages following the WHO translation guidelines for assessment instruments, which included a forward translation, a targeted back-translation, a review by a bilingual expert group and a detailed translation report [21]. Face-to-face interviews were conducted at the respondents' homes by trained interviewers. Further details of the design and methods have been published elsewhere [22].

Ethical approvals from the following institutions were obtained: Ethics Review Committee, World Health Organization; Ethical Committee, Ghana Medical School, Accra, Ghana; Institutional Review Board, International Institute of Population Sciences, Mumbai, India; Ethics Committee, National Institute of Public Health (INSP), Cuernavaca, Mexico; and Ethics Committee, OPM (School of Preventive and Social Medicine), Russian Academy of Medical Sciences, Moscow, Russia. Written informed consent from each participant was also obtained.

### Study instruments

Presence of 12-month depressive episode was based on the International Classification of Diseases, Tenth Revision (ICD-10) diagnostic criteria for research for depressive episodes [23], and was derived from an algorithm that took into account respondents reporting symptoms of depression during the previous 12 months [24]. Individuals were additionally asked whether they had ever been diagnosed with depression by a health professional or had ever taken medication or received some other treatment (e.g. psychotherapy). Participants who experienced 12-month depression in Wave 1 but did not report having experienced lifetime depression in Wave 0 were defined as the incident depression group. In turn, respondents who reported 12-month depression at both waves were defined as the persistent depression sample.

Alcohol consumption was assessed by the question, “Have you ever consumed a drink that contains alcohol (such as beer, wine, spirits, etc.)?” Those who answered “no” were categorized as “never” drinkers. For those answering “yes”, a separate question was asked about how many drinks of any alcohol beverage they had taken on each day of the previous week. Heavy drinkers were defined as those who consumed at least five (for men) or four (for women) standard alcoholic drinks per day on at least one day in the week before the interview [25]. Non-heavy drinkers were defined as any respondent who have ever consumed alcohol but were not heavy drinkers.

Current smoking was defined as a binary variable indicating whether the respondent currently smoked any tobacco product. Current smokers were categorized into “non-daily smokers” and “daily smokers” if the respondent reported to be an occasional smoker or to smoke on a daily basis, respectively. Although the use of smokeless tobacco is known to cause health problems, its use was only collected in Wave 0 in the countries where smokeless tobacco was a relevant issue, such as India. For data comparability reasons with the rest of the countries, individuals who only used smokeless tobacco were excluded from analyses.

Physical activity was measured with the International Physical Activity Questionnaire (IPAQ): short form [26] in Wave 0 and with the Global Physical Activity Questionnaire (GPAQ) in Wave 1 [27]. Physical activity scores were categorized into high, moderate and low physical activity [26, 27].

Presence of at least one of the following chronic physical conditions was obtained: arthritis, asthma, angina pectoris, and diabetes. Individuals were considered to have the health condition when they had been diagnosed with it at some point and had taken medication or some other treatment for it. Additionally, respondents were also identified to have arthritis, asthma or angina if they reported the presence of the core symptoms of the condition during the previous 12 months using diagnostic algorithms that have been used previously [28].

Body mass index (BMI) was used to assess underweight, normal weight, overweight and obesity. Using the standard WHO definition, BMI was categorized as <18.5 kg/m^2^ (underweight), 18.5-24.9 kg/m^2^ (normal weight), ≥25.0 kg/m^2^ (overweight / obesity).

Health status was assessed with a set of health-related questions covering eight health domains: mobility, self-care, pain and discomfort, cognition, interpersonal activities, vision, sleep and energy, and affect [29]. For each question, the responses were recorded on a 5-point scale ranging from no difficulty/problem to extreme difficulty. An overall health status score based on these health-related questions was obtained using a Rasch model [30]; the health status scores ranged from 0 to 100, with higher scores indicating better health.

Socio-demographic information, including age, gender, education level, employment, and country-specific household income were reported at the beginning of the interview. The country-specific household income was estimated for each respondent using an assets-based approach [31]. The index was further divided into quintiles within each country, where quintile one represented the poorest wealth quintile and quintile five the richest.

### Data analysis

Firstly, the percentages of people with incidence and persistence of depression were calculated. Incidence was calculated dividing the number of people with 12-month depression in Wave 1 by the number of people without lifetime depression in Wave 0. Persistence of depression was calculated dividing the number of people with 12-month depression in Wave 1 by the number of people with 12-month depression in Wave 0.

General characteristics of respondents with incident depression and persistent depression in Wave 1 were described. Pairwise comparisons between these groups (people with incident depression and people with persistent depression) were undertaken by a *t*-test for continuous variables and chi-square tests for categorical variables. Cohen’s d and Cramer's V were calculated as effect sizes for significant differences found in t-tests and chi-square tests, respectively.

To check whether baseline characteristics were associated with missing data on 12-month depression in Wave 1, pairwise comparisons between participants with and without missing values were undertaken by t-tests and chi-square tests for continuous and categorical variables, respectively. Effect sizes were also calculated in case of significant differences.

A first logistic regression analysis including 12-month depression in Wave 1 as an outcome variable was undertaken to estimate whether baseline unhealthy lifestyles were predictive of incident depression. For this first analysis, people with lifetime depression at Wave 0 were excluded, leaving an analytical sample of 6349 persons. Unhealthy lifestyles were included as the main predictors. This analysis was also controlled for demographics, presence of a physical chronic condition, BMI, general health status and country.

Finally, a second logistic regression analysis, but including people with 12-month depression in Wave 0, was undertaken to estimate factors longitudinally associated with persistent depression.

Odds Ratio (OR) and 95% confidence intervals (95% CI) were displayed for the logistic regressions. Results were considered statistically significant when *p* ≤ 0.05. Statistical analyses were conducted using Stata version 11.

## Results

### General characteristics of the sample

Table 1 includes baseline general characteristics of respondents with persistent depression, with incident depression in Wave 1 and pairwise comparisons between these two groups. A total of 594 adults out of the 5970 participants without lifetime depression in Wave 0 (10.0%) were classified as having incident depression in Wave 1. A total of 219 adults out of the 1267 participants who experienced 12-month depression in Wave 0 (26.4%) were classified with depression at both time points (persistent depression). In comparison to adults with incident depression in Wave 1, those with persistent depression were more frequently women, suffered more frequently from at least one chronic physical health condition and experienced lower health status. No significant differences were found in the baseline frequencies of smoking, physical activity and heavy alcohol use between people with incident depression in Wave 1 and people with persistent depression in Wave 1 (Table 1).

**Table 1:** Sample characteristics by depression status in SAGE Wave 1

Baseline variables	Persistent depression	Incident depression	*p* ^1^ (E.S)^2^
Age n (%) Mean (SD)	51.38 (14.77)	50.24 (15.39)	0.82
Women, n (%)	161 (75.23)	395 (66.50)	0.018 (0.08)
Unemployed, n (%)	73 (50)	180 (40.91)	0.060
Country, n (%)			0.37
Mexico	80 (37.38)	203 (34.18)	
Ghana	4 (1.87)	25 (4.21)	
Russia	6 (2.80)	21 (3.54)	
India	124 (57.94)	345 (58.1)	
Household income, n (%)			0.21
1^th^ & 2^nd^ quintile	89 (41.59)	218 (36.7)	
3^rd^, 4^th^ & 5^th^ quintile	125 (58.41)	376 (63.30)	
Education, n (%)			0.65
Less than primary	92 (42.99)	244 (41.22)	
At least primary completed	122 (57.01)	348 (58.78)	
Health conditions, n (%)			<0.001 (0.23)
None	56 (30.11)	293 (55.81)	
At least one	130 (69.89)	232 (44.19)	
Global health score, Mean (SD)	60.15 (10.01)	70.10 (14.22)	<0.001 (0.75)
Alcohol n (%)			0.98
Never drinkers	151 (92.07)	449 (92.20)	
Current drinkers	10 (6.10)	31 (6.35)	
Heavy drinkers	3 (1.83)	8 (1.64)	
Current smoking n (%)			0.51
No current smokers	137 (65.24)	400 (69.57)	
No-daily smokers	16 (7.62)	37 (6.43)	
Daily smokers	57 (27.14)	138 (24.0)	
BMI n (%)			0.72
Normal weight	119 (71.69)	355 (70.44)	
Underweight	21 (12.65)	76 (15.08)	
Overweight/obese	26 (15.66)	73 (14.48)	
Physical exercise n (%)			0.25
Highly active	135 (63.08)	381 (64.14)	
Moderately active	23 (10.75)	84 (14.14)	
Inactive	56 (26.17)	129 (21.72)	

^1^ *p*-value associated to differences among sets using Chi-squared test (categorical variables) or *t* test (continuous variables)

^2^Effect size measure. Cramer’s V for Chi-squared tests and Cohen’s d for *T* test

### Analyses with missing values on the outcome variable

Older people in Wave 0 (*t*(7896) = 3.33; *p* < 0.001; d = 0.16), participants from Ghana (*χ* ^2^ (3) = 13.23(3), *p* = 0.004, V = 0.05) and people who suffered from any chronic health condition in Wave 0 (*χ* ^2^(1) = 208.47, *p* = 0.004, V = 0.05) showed higher missing values on 12-month depression in Wave 1. However, Cramer's V was lower than 0.20 in all cases, indicating a very low effect size according to Cohen’s guidelines [32].

### Unhealthy lifestyles and incidence of depression

Table 2 shows the baseline risk factors for incident 12-month depression in Wave 1. Results revealed that older persons and respondents with at least one chronic health condition were at higher risk for incident depression in Wave 1. Regarding the unhealthy patterns, non-daily and daily smokers were at higher risk for incident depression in Wave 1, compared with never-smokers. The risk of an incident depression in the follow-up was higher among underweight adults in comparison with the normal BMI range group. Similarly, the presence of at least one chronic health condition in baseline was related to the depression in Wave 1. Health status, gender, employment status, obesity/overweight, alcohol consumption patterns and physical activity levels were not related to incident depression in Wave 1.

**Table 2:** Odds ratios for the association between baseline risk factors and incident and persistent depression in SAGE Wave 1 (2007/10)

Baseline risk factors^a^	Persistent depression		Incident depression	
	O.R. (95% CI)	*p*	O.R. (95% CI)	*p*
Sex (ref. men)	0.88 (0.49, 1.59)	0.69	1.15 (0.84, 1.57)	0.37
Age	0.99 (0.98, 1.01)	0.61	1.02 (1.00, 1.02)	<0.001
Unemployed (ref. employed)	1.25 (0.75, 2.09)	0.39	0.75 (0.63, 1.11)	0.22
Education (ref. less than primary)	0.82 (0.49, 1.38)	0.47	0.78 (0.59, 1.04)	0.087
Household income (ref. quintile 3^rd^, 4^th^ and 5^th^)	0.61 (0.39, 0.94)	0.027	0.87 (0.68, 1.12)	0.29
Current smokers (ref. not-current smoker)				
Non-daily smokers	0.38 (0.05, 2.99)	0.36	2.06 (1.18, 3.62)	0.011
Daily smokers	0.93 (0.55, 1.57)	0.80	1.46 (1.09, 1.97)	0.012
Alcohol consumption (ref. never drinkers)				
Non-heavy drinkers	0.98 (0.36, 2.61)	0.96	0.93 (0.57, 1.52)	0.78
Heavy drinkers	4.72 (1.03, 21.72)	0.040	1.59 (0.67, 3.75)	0.29
Physical inactive (ref. high active)				
Moderate physical activity	0.59 (0.23, 1.48)	0.26	0.97 (0.66, 1.42)	0.89
Low physical activity	2.11 (1.24, 3.59)	0.006	1.23 (0.89, 1.68)	0.20
BMI (ref. normal range)				
Underweight	0.64 (0.36, 1.15)	0.14	1.45 (1.07, 1.96)	0.016
Overweight/obese	1.48 (0.75, 2.89)	0.26	0.99 (0.68, 1.00)	0.97
Chronic condition (ref. none)	1.28 (0.78, 2.10)	0.32	1.32 (1.02, 1.72)	0.034
Global health	0.92 (0.96, 1.00)	0.11	0.99 (0.98, 1.00)	0.14

^a^Analyses were controlled by country (Mexico was the reference category)

### Unhealthy lifestyles and persistence of depression

Table 2 also reports risk factors for experiencing 12-month depression at both time points. Participants with medium-high household income were at lower risk for persistent depression in comparison with those who ranked in the two lower household income quintiles. Regarding unhealthy lifestyles, baseline heavy drinkers were at higher risk for persistent depression in comparison with abstainers. In addition, people with low physical activity at baseline were at higher risk for persistent depression in comparison with those having high physical activity level. Baseline health status, presence of chronic diseases, BMI and smoking were not related to persistent depression.

## Discussion

To our knowledge, this is one of the few studies to analyze longitudinal relationships between unhealthy lifestyles and depression using data from four emerging countries: India, Chana, Mexico and Russia.

Firstly, baseline frequencies of unhealthy lifestyles between people who developed depression in Wave 1 and people with depression in both waves were not significantly different. These results are not congruent with a previous study showing a dose–response relationship between history of depression and the higher prevalence of smoking, physical inactivity and heavy drinking. [4] Nonetheless, this latter study was cross-sectional and did not analyse chronicity. The fact that unhealthy lifestyles were similar between people with depression and people who prospectively developed depression did not support the hypothesis of “self-medication” of depressive symptoms. In turn, these results might be in line with the existence of common vulnerable factors between depression and unhealthy lifestyles. However, our results do not allow us to completely reject the self-medication hypothesis. People who developed depression in Wave 1 might have experienced subthreshold symptoms of depression in Wave 0, which can be also associated with distress and disability [33]. Further studies with weighted prevalence estimations are necessary to confirm these results.

Regarding the factors associated with the onset of depression in Wave 1, the results show that baseline daily smoking was associated with depression in Wave 1. This finding is in line with the previous literature collected in high income countries [10, 34]. One study reported that the relationship between tobacco use and depression might be both direct and indirect [10]. Several shared genetic factors have been found between depression and tobacco use [35]. In addition, some authors have suggested that specific inflammation markers possibly triggered by tobacco use and that are also correlated with the onset of depression could also explain the relationship between tobacco and depression [36]. However, the study of the mediating role of inflammation markers between unhealthy lifestyles and depression needs to be further explored [12]. In addition, other authors in China found that smokers were at lower risk for depression than never smokers [13]. These contradictory findings might be explained by methodological differences in the measurement of tobacco use and in the control for confounders since this last study did not consider the effect of income or the presence of chronic health conditions. One study suggested that there are also environmental factors in the relationship between tobacco and depression and that their importance might vary based on age participants [35]. Further longitudinal studies should be conducted in emerging countries to confirm whether there are specific cultural factors explaining the relationship between tobacco use and depression. Although our study is longitudinal and we restricted our analyses to people who neither experienced 12 months depressive symptoms in baseline nor were previously diagnosed with depression, reversed causality (i.e. depression leading to tobacco use) cannot be completely ruled out, since smokers in baseline could have experienced an undiagnosed depressive episode prior to Wave 0 which could make them more likely to smoke [4].

The present study also found that the non-daily smoking group was more likely to experience depression in Wave 1 than the daily smoking group in comparison with never smokers. This finding is not in accordance with the previous literature reporting that heavy smokers are more likely to be depressed than light smokers [34]. However, another study suggested that relationship between higher frequency of tobacco use and depression was only significant in younger ages [37]. On the other hand, non-daily smoking has been correlated to depression, specifically in women [38]. Further studies should confirm whether non-daily smokers are the highest risk group for depression, including gender-and-age-specific analyses.

Nearly 80% of the world’s tobacco users are located in low-and-middle-income countries [39]. Our results suggest that daily and no daily smokers were more likely to become depressed over time. Therefore, health care providers in emerging countries can benefit from this line of study if further research can check whether smoking cessation can reduce depression in emerging countries as it has been reported in high income countries [40].

Additionally, our analyses also examined whether people with depression and unhealthy lifestyles in Wave 0 were more likely to continue having depression in Wave 1. Heavy drinkers that were depressed in baseline were more likely to still be depressed in the follow-up. This is in line with studies conducted in higher-income countries where heavy drinking has been associated with both recurrent and chronic depression [41, 42]. However our study did not consider the temporal sequence between heavy drinking and depression therefore, we cannot assure whether heavy alcohol drinking was a risk factor for persistent depression or if on the contrary, it was chronic depression that increased the likelihood of becoming heavy drinker [5].

Similarly, depressed participants with lower physical activity were more likely to also be depressed in the follow-up. This finding is congruent with previous studies [43]. Some evidence supports increased physical activity levels for those with depression to reduce residual symptoms and prevent further relapses [44]. However, this finding might also be explained by the fact that people with low physical activity might also suffer from higher severity of depression, which has also been related to a worse course of depression [45]. Our study was not able to assess when physical activity and depression emerged, consequently we cannot assure whether lower physical activity was a risk factor for chronicity of depression or whether people with chronic depression are persistently physical inactive [5]. Further studies should confirm whether a chronic course of depression is associated with a drop in physical activity, including time-varying factors models.

Finally, in contrast to previous evidence, our study did not find that depressed smokers were more likely to continue having depression in Wave 1 [8]. Further studies should check whether a more chronic course of depression might increase tobacco use [5] and also whether persistent smokers are more likely to experience acute episodes of depression.

The main strengths of the present study are the use of the same methodology and the same evaluation instruments in a large longitudinal study including four different countries and the control for important confounders in the statistical analyses. Nevertheless, our results also need to be interpreted taking into account some limitations. The diagnosis of depression was made using fully structured, trained, lay interviewers rather than clinician-administered interviews. Nevertheless, the clinical SCID-CIDI reappraisal interviews have shown a good concordance in the Diagnostic and Statistical Manual of Mental Disorders diagnoses (DSM) [46]. Another limitation of the present work is the lack of a rating scale measure to assess the severity of depression. We also acknowledge that some people who screened negative for lifetime depression in Wave 0 might have experienced a previous depressive episode that may have gone unrecognized or untreated. Nonetheless, our study did not aim to estimate the impact of unhealthy lifestyles on incidence of first-time occurrences of depression, but rather the role of these modifiable factors on the subsequent development of depression among those who had neither a depressive episode in the 12 months preceding the baseline assessment nor a previous lifetime depression diagnosis. In addition, the data did not allow us to differentiate whether 12-month depression in Wave 1 was really a recurrent episode with an interim period of remission, or a non-remitting course of the 12-month depressive episode collected in Wave 0. Further studies, including more frequent measurement or questions about the period between the two waves, could address the role of unhealthy behaviours in the clinical course of depression, differentiating between non-remitting or recurrent depressive episodes. Finally, three baseline factors were associated with missing information. However, the effect sizes of these differences were small.

## Conclusions

The present study provides evidence collected in four emerging countries about the positive relationships between some unhealthy lifestyles and depression. In this study, daily and non-daily smokers were both more likely to become depressed over time. The results of this study together with the studies undertaken in high income countries might support the idea of common mechanisms explaining the relationship between tobacco use and depression across countries. However, further studies are still necessary to understand this relationship. In addition, this study also suggests that depressed people with low physical activity and with heavy drinking patterns were both more likely to continue having depression over time. Further studies should confirm whether unhealthy lifestyles are risk factors for a chronic course of depression or the other way around. Health care providers from emerging countries should consider unhealthy lifestyles in patients with depression. Further studies could check whether the management of healthy lifestyles might also help to prevent and improve depression in emerging countries.

## Abbreviations

BMI: Body mass index
SAGE: Study on Global AGEing and Adult Health
WHO: World Health Organization
